# Morphological, pathogenic and genetic diversity in *Diplodia seriata* associated with black rot canker of apple in India

**DOI:** 10.1038/s41598-023-42534-y

**Published:** 2023-09-21

**Authors:** Arif Hussain Bhat, M. D. Shah, B. A. Padder, Zahoor Ahmad Shah, Eajaz Ahmad Dar, U. Fayaz, Manjeet Singh Nain, M. Ajmal Ali, Fahad M. Al-Hemaid, Piotr Stępień, Mohamed A. A. Ahmed, Ehab A. A. Salama

**Affiliations:** 1 ARSSSS-Pampore, SKUAST-K, Shalimar, Srinagar, J&K India; 2 Division of Plant Pathology, SKUAST-K, Shalimar, Srinagar, J&K India; 3 Krishi Vigyan Kendra Ganderbal, SKUAST-K, Shalimar, Srinagar, J&K India; 4 Division of Agricultural Extension, ICAR-IARI, New Delhi, 110012 India; 5 Department of Botany and Microbiology, College of Science, King Saud University, 11451 Riyadh, Saudi Arabia; 6 Department of Plant Nutrition, Institute of Soil Science, Plant Nutrition and Environmental Protection, Wroclaw University of Environmental and Life Sciences, ul. Grunwaldzka 53, 50-357 Wroclaw, Poland; 7 Plant Production Department (Horticulture - Medicinal and Aromatic Plants), Faculty of Agriculture (Saba Basha), Alexandria University, Alexandria, 21531 Egypt; 8 School of Agriculture, Yunnan University, Chenggong District, Kunming, 650091 Yunnan China; 9 Department of Agricultural Botany (Genetics), Faculty of Agriculture Saba Basha, Alexandria University, Alexandria, Egypt; 10 Department of Plant Biotechnology, Centre for Plant Molecular Biology and Biotechnology, TNAU, Coimbatore, 641003 India

**Keywords:** Plant sciences, Plant immunity, Effectors in plant pathology

## Abstract

Apple cankers are extremely destructive diseases threatening the global apple industry through direct and indirect losses. The population structure of the pathogens is of paramount significance for the development of efficient management strategies. Therefore, phenotypic, pathogenic, and genetic diversity of *Diplodia seriata* causing black rot canker of apple was investigated in this study. All the isolates were included for investigating the in vitro mycelial growth, conidial dimensions, and pathogenic variability on two-year-old potted apple seedlings. The ISSR approach was used to investigate the molecular diversity of *D. seriata*. Mycelial growth rates were found to vary significantly amongst the isolates; however, there were no major variations seen between the different geographical groupings of isolates. Pathogenicity tests revealed variations in the size of cankers among the isolates indicating the presence of virulence variability. The isolates were segregated into three virulence groups based on canker length. The Bayesian analyses of ISSR data divided the isolates into two genetic clusters. The genetic clustering of the isolates revealed no relationship with geographical origin of the isolates. Furthermore, no direct relationship of genetic clustering was observed with morphological or pathogenic variability. The ISSR primers revealed very high level of variability in *D. seriata*; however, no distinct populations of the pathogen existed which is an indication of high level of gene flow between the diverse geographical populations. According to our knowledge, this is the first thorough investigation on the diversity of *D. seriata* associated with apple black rot canker in India.

## Introduction

Apple (*Malus domestica*) is the oldest temperate fruit which is very popular around the world and assumes an excellent global market. It has varied size, shape, color, taste, texture, aroma, and nutritive value fulfilling the needs and desires of global population. Apple is cultivated in temperate and subtropical areas and ranks fourth globally in production after citrus, grapes, and banana^1^.

*Diplodia seriata* has been reported on diverse plant genera due to its cosmopolitan and plurivorous nature. The fungus has been reported from 35 plant species^2^. *Diplodia seriata* causes canker, dieback, fruit rot and leaf spots on economically important forest and horticultural species^3^. A few examples of the diseases and first reports caused by *Diplodia seriata* are branch canker and *Pinus pinea* decline^4^, twig and branch canker in mulberry^5^, spring bud mortality, leaf chlorosis, fruit rot and trunk dieback on grapevines ^6,7^, first report of fruit rot of loquat^8^, first repot of canker and dieback on hawthorn^9^ and first report of dieback disease on cedars^10^. Nevertheless, Systematic Mycology and Microbiology Laboratory Fungal Database (ARS, USDA) indicates that the host range for *D. seriata* now is significantly wider, with over 200 species and 130 genera of herbaceous and woody plants functioning as hosts^11^. It has been reported to produce different symptoms like cankers, dieback of shoots, fruit rot, and leaf spots on a wide category of horticultural crops^12^. *Diplodia seriata* (syn. *Botryosphaeria obtusa*) is an important pathogen of apples inciting diseases like frog-eye leaf spot, black rot, dieback, and cankers^13^. The occurrence of black rot on apple fruit was first reported by Peek from New York in 1879, whereas its leaf spot phase was reported by Alwood in 1892 and its destructive canker phase (Black rot canker) was reported by Paddock in 1899^14^. Mundkur and Kheswala^15^ reported the smoky blight (*Sphaeropsis malorum* Berk.—synonym of *D. seriata*) from imported apple plants in Mysore, India. Infection occurs through wounds or by direct penetration through natural openings^16–18^ followed by colonization of the healthy tissues.

The smoky or black rot canker develops on branches, limbs and trunks as small, sunken, reddish-brown lesions which rapidly enlarge lengthwise, become elliptical, develop alternate rings, turn smoky and partially or completely girdle the affected part of the plant. While superficial roughening of the bark is typical, in certain instances the bark is destroyed up to the wood and totally shattered^19,20^. However, Singh^21^ recorded the development of cracks along the lesion margins; thus, temporarily restricting canker elongation. In advanced stages the bark becomes loose and gets separated from the wood. The initial symptoms are identified by brown discoloration of the bark on branches and twigs followed by leaf chlorosis. The bark cracks as the disease spreads further into the timber, revealing the reddish-brown discolored wood below. Internal browning of the woody tissues is seen on splitting the infected twigs along the axis. The spurs, twigs and branches above the cankered portion are killed without developing any canker symptoms. On the bark of diseased or dead branches and twigs, many black pycnidia of the fungus may be seen^22^.

Pathogenicity of *D. seriata* has been evaluated on shoots, fruits, and leaves of apple in some countries^23,24^. The experimental data on the pathogenic nature of *Diplodia* spp. on woody plants is controversial^25^. The studies on the pathogenicity of different *D. seriata* strains on various hosts and in different regions have shown inconsistent results^26^. In England and New Zealand, it is considered as secondary and weak pathogen of apples^27^, while reported as highly pathogenic on apples in the USA^14,28,29^. Similarly, contrary reports on the pathogenic nature of *D. seriata* on grapevines have been reported from different parts of the world^12^. Although different symptoms like dark vascular discoloration, extended necrotic lesions on shoots and formation of cankers have been linked to pathogenicity of *Botryosphaeriaceae* species^30^, pathogenicity and virulence of the canker causing fungi is normally estimated by measuring their capability to develop brown necrotic lesions beneath the bark and the lesion size, respectively^12^. Larignon^31^ studied the pathogenic variability in *D. seriata* and divided the isolates into four virulence groups based on variations in lesion size. The variations in pathogenicity level of *D. seriata* isolates on grapevines has been reported in earlier studies^6,30,32^. Savocchia^33^ also recorded pathogenic variability in *D. seriata* isolates based on intraspecific variations within this fungus.

For genetic diversity analysis, inter-simple sequence repeat (ISSR) or random amplified microsatellite (RAMS) technique^34,53^ have been proved effective^35^. The relative participation of telomorphic and anamorphic forms contributes to genetic diversity of a species. For *Botryosphaeriaceae* species, the telomorphs are rarely found in nature^6^ and the reproduction is mainly asexual under natural conditions^30,36^. However, exchange of genetic material can take place between compatible genotypes by parasexual recombination^37^. In Spain the genetic diversity of *D. seriata* isolates derived from grapevine was studied by Martín^38^ using AFLP markers but no data on pathogenic diversity was provided. Elena^12^ studied phenotypic, pathogenic, and genetic diversity of *D. seriata* on grapevine and reported high level of diversity within populations but no relationships among the genetic grouping and the virulence of different isolates was observed. Investigations on genetic variance between the populations of *Lasiodiplodia theobromae* revealed very low gene flow and genotypic diversity between the geographic populations, and increased gene flow and genotypic diversity among the pathogen populations resident on different host plants^39^. Genetic and virulence variability in the isolates of *Neofusicoccum parvum* was reported by Baskarathevan^40^. Nevertheless, no association was found between genetic clustering and isolate virulence. Genetic diversity existed between 66 isolates of *Neofusicoccum luteum* belonging to different pathogenic groups; however, the pathotype–genotype relationships were not found^41^. The genetic diversity studies on *Togninia minima* reported no significant differences in virulence between the genetic groups^42^. To the best of our knowledge, no comprehensive studies on the combined genetic, phenotypic, and pathogenic characterization on the isolates of *D. seriata* associated with black rot canker of apple have been undertaken.

The main purpose of the present investigation was to acquire more satisfactory comprehension of the phenotypic, pathogenic, and genetic diversity of *D. seriata* responsible for black rot canker of apple trees in India. The pathogenicity assays conducted on young potted apple seedlings were used to estimate the pathogenic variability, whereas mycelial growth rates and conidial dimensions were used for phenotypic characterization. The final aim of our investigations was to verify the potential existence of distinct populations of *D. seriata* in different geographical regions based on combined morphological, pathogenic, and genetic studies, and the expansion of our understanding regarding *D. seriata* which may help us in understanding the pathogenic nature of *D. seriata* in apple and will have an impact on the efficient disease management strategies.

## Results

### Morphological variability

#### Mycelial growth

**Figure 1:**
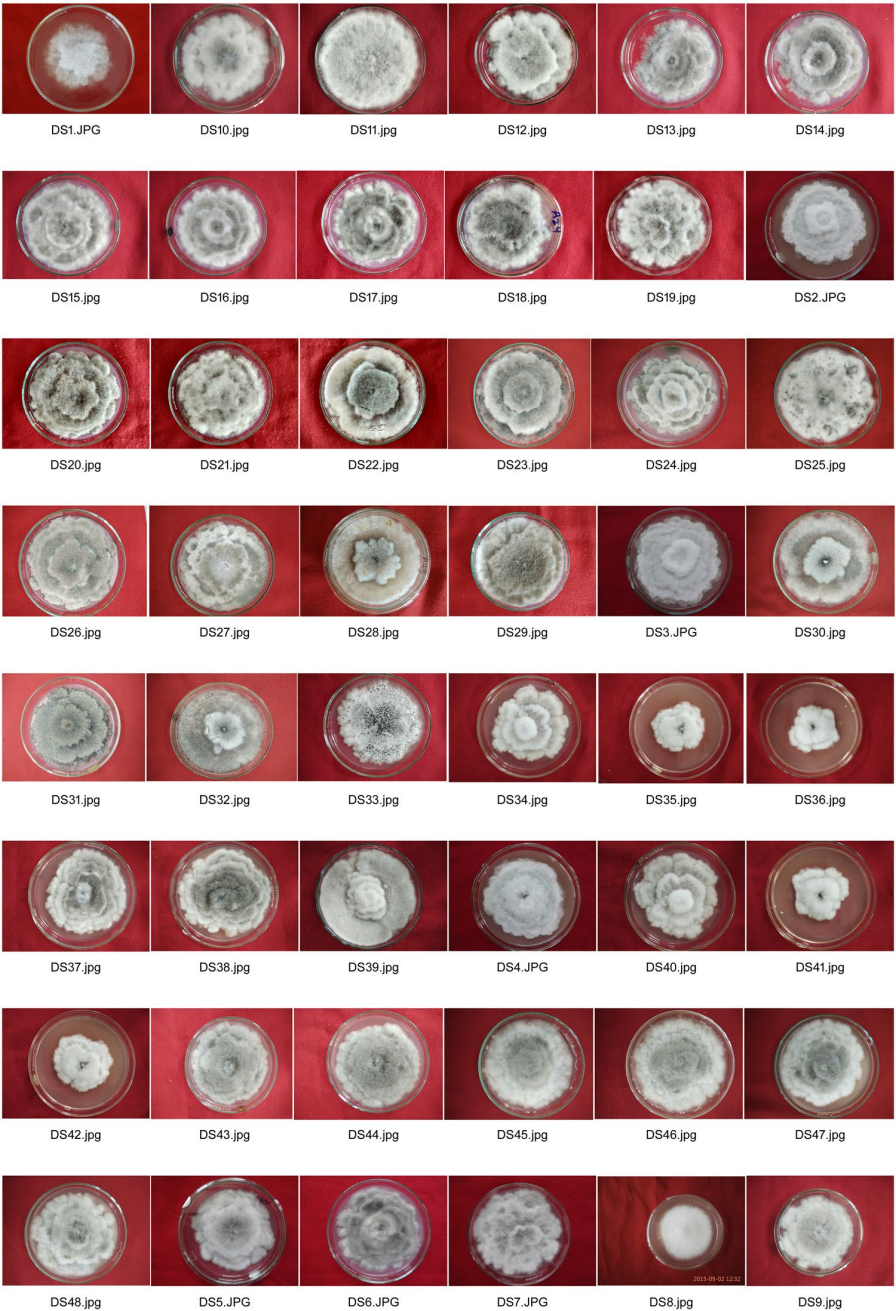
Relative radial mycelial growth of the isolates of *D. seriata* on PDA.

The radial mycelial growth of isolates of *D. seriata* was assessed by growing the isolates on PDA. All the isolates were involved in this investigation (Fig. 1). The isolates showed significant differences in mycelial growth rates (*P* < 0·001). The mean mycelial growth rate for all the isolates with 95% confidence interval was 2.66 ± 0.87 cm, with minimum and maximum mycelial growth rates as 2.13 cm and 3.18 cm, respectively. The variation in radial mycelial growth of the isolates had no relation with the grouping of isolates based on geographical origin as well as with virulence groups. The statistically significant differences in growth rates were not observed among the geographical groups of isolates (Table 1).

**Table 1:** Mycelial growth rate and canker size produced by geographical groups of *D. seriata* isolates on potted apple saplings (cultivar Red delicious).

Geographical groups	Mycelial growth rate (cm)			Canker length on saplings (cm)		
	Mean	CI	Sig	Mean	CI	Sig
PL	2.60	0.17	a	10.08	1.43	a
SH	2.66	0.17	a	9.67	0.93	a
BR	2.70	0.17	a	10.85	1.61	a
GN	2.66	0.20	a	11.61	1.60	a
Control	–	–	–	0.90	0.18	b

*PL Pulwama, *SH* Shopian, *BR* Baramullah, *GN* Ganderbal, *CI* Confidance interval.

#### Conidial characteristics

All the isolates of *D. seriata* under experimental conditions produced conidia on oatmeal agar medium. The mean conidial size of the isolates grouped based on geographical origin was not found to present considerably different values in length, width, and length:width ratio between the groups. Among the geographical groups, the absolute differences calculated between the group means were 0.87 µm between group 1 and group 2 (group 1, 22.91 ± 0.61 µm; group 2, 22.04 ± 1.22 µm), 0.23 µm between group 1 and group 3 (group 3, 23.14 ± 0.44 µm), 0.81 µm between group 1 and group 4 (group 4, 23.72 ± 0.65 µm), 1.10 µm between group 2 and 3, 1.68 µm between group 2 and 4, 0.58 µm between group 3 and 4 in length. Similarly the absolute differences in means of width among the geographical groups were as; 0.14 µm for group 1 and 2 (group 1, 10.58 ± 0.31 µm; group 2, 10.72 ± 0.44 µm), 0.25 µm between group 1 and 3 (group 3, 10.33 ± 0.36 µm), 0.50 µm between group 1 and 4 (group 4, 10.08 ± 0.15 µm), 0.39 µm between group 2 and 3, 0.64 µm between group 2 and 4, 0.25 µm between group 3 and 4, respectively. The mean L:W ratio of the geographical groups was 2.18 ± 0.08 for group 1, 2.07 ± 0.12 for group 2, 2.26 ± 0.07 for group 3 and 2.36 ± 0.07 for group 4 respectively (Table 2). The mean length: width ratio for all the isolates was 2.22 ± 0.04. For all the isolates, the min., maximum and mean ± 95% confidence interval values were in the following range: length (14.13) − 22.95 ± 0.77 μm − (29·24) × width (8.91) − 10.43 ± 0.32 μm − (13·75).

**Table 2:** The conidial dimensions of the geographical groups of *D. seriata* isolates.

Groups	Group 1	Group 2	Group 3	Group 4
Length	22.91 ± 0.61 µm*	22.04 ± 1.22 µm	23.14 ± 0.44 µm	23.72 ± 0.65 µm
Width	10.58 ± 0.31 µm	10.72 ± 0.44 µm	10.33 ± 0.36 µm	10.08 ± 0.15 µm
L:W ratio	2.18 ± 0.08	2.07 ± 0.12	2.26 ± 0.07	2.36 ± 0.07

*Mean ± 95%CI.

### Pathogenic variability

**Figure 2:**
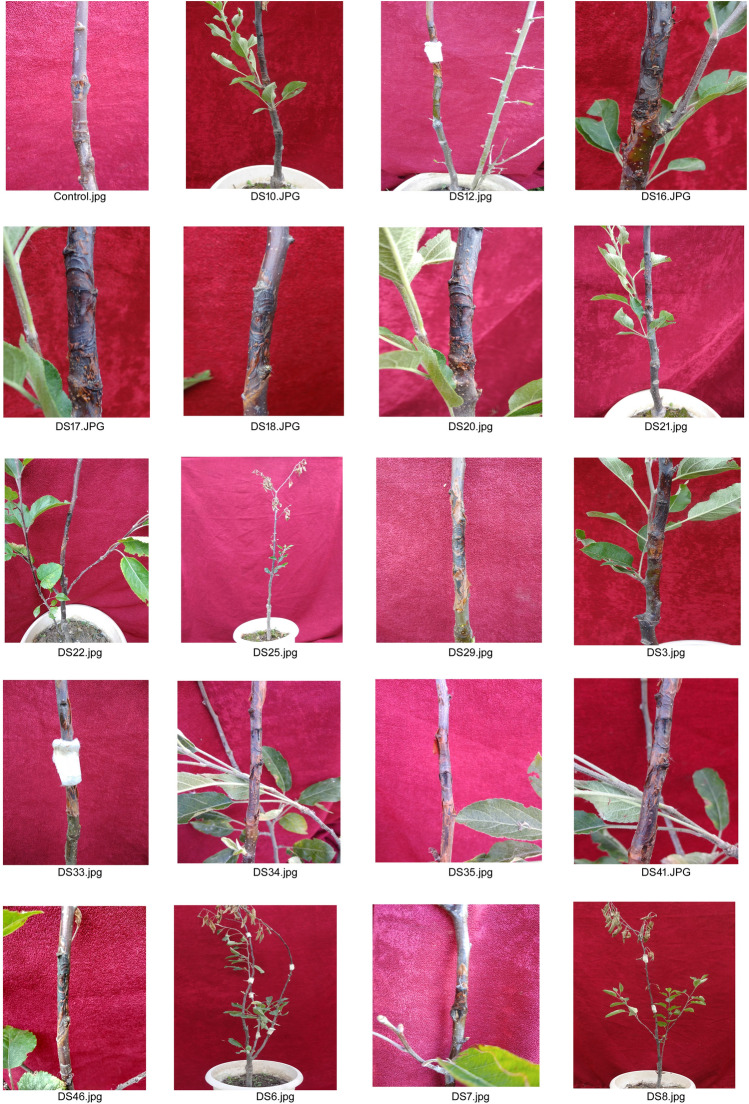
Prominant canker symptoms produced by *Diplodia seriata* isolates on potted apple plants cv. Red Delicious after 3 months of inoculation.

**Figure 3:**
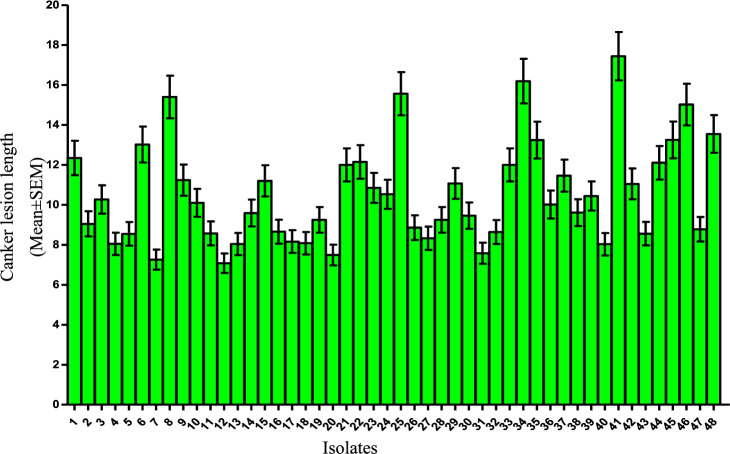
Mean canker size of *D. seriata* isolates caused on the apple cultivar red delicious. Bars represent the retransformed means, and the error bars are representative of upper and lower 95% confidence intervals.

All the isolates of *D. seriata* were tested for pathogenicity on potted apple seedlings. Each of the isolates produced smoky canker symptoms characterized by water soaked, yellow to brown discoloration at the site of incision. The lesion showed upward and downward extension, girdling and rupturing of bark. Characteristic concentric ring development was also observed in the lesions (Fig. 2). During the investigation, significant differences in canker length between the isolates were observed in statistical analysis. All the isolates produced cankers considerably larger than control (Fig. 3) with mean lesion length varying from 7.08 cm (isolate 12) to 17.43 cm (isolate 41) and an overall mean lesion length of 10.35 cm. In each geographical group of isolates the mean lesion length was considerably longer than that of control but no considerable differences were observed amongst the groups (Table 1). The mycelial growth rates recorded under in vitro conditions and the length of canker lesions recorded during pathogenicity tests were not found to have any relationships. The overall average positive re-isolation from the inoculated twigs was 2.94 out of 3 twigs, i.e., 98% re-isolation frequency; thus, proving that all the isolates were pathogenic in nature.

The isolates exhibited a considerable variability in incubation periods during pathogenicity tests. Based on incubation period the isolate were divided into three groups. The first group comprised of isolates for which the mean incubation period was 31.93 ± 2.60 days to develop canker symptoms on the inoculated plants. A total of 37.50% isolates belonged to this group. The second group comprised of 35.41% isolates which exhibited an incubation period of 44.59 ± 2.61 days, while as the third group comprising of 27.08% isolates was found to show the longest incubation period of 59.79 ± 2.49 days (Table 3).

**Table 3:** Grouping of isolates based on incubation period.

Groups	G1		G2		G3	
Percent isolates	37.50%		35.41%		27.08%	
S. no.	Isolates	Incubation period (days)	Isolates	Incubation period (days)	Isolates	Incubation period (days)
1	DSS2	31.33 ± 1.53*	DSS1	47.33 ± 2.08*	DSS6	56.33 ± 1.53*
2	DSS4	29.67 ± 1.53	DSS3	50.67 ± 1.53	DSS8	59.67 ± 1.53
3	DSS5	35.33 ± 1.53	DSS9	42.00 ± 1.00	DSP9	61.67 ± 1.53
4	DSS7	37.00 ± 2.00	DSS10	45.33 ± 1.15	DSP10	61.33 ± 0.58
5	DSS11	30.33 ± 2.08	DSP2	45.33 ± 1.53	DSG1	64.67 ± 0.58
6	DSS12	30.00 ± 1.00	DSP3	47.33 ± 1.15	DSG9	56.00 ± 1.00
7	DSP1	35.33 ± 1.15	DSP7	42.33 ± 0.58	DSG10	57.67 ± 2.08
8	DSP4	33.67 ± 1.53	DSP11	45.33 ± 2.52	DSG11	60.67 ± 2.52
9	DSP5	30.33 ± 2.52	DSP12	48.33 ± 1.15	DSB5	57.33 ± 1.53
10	DSP6	29.00 ± 1.00	DSG4	42.67 ± 1.53	DSB8	60.00 ± 2.00
11	DSP8	34.33 ± 1.53	DSG5	42.00 ± 2.00	DSB9	62.33 ± 2.08
12	DSG2	31.67 ± 1.53	DSG6	43.00 ± 1.73	DSB10	60.33 ± 2.52
13	DSG3	36.00 ± 1.00	DSG12	42.00 ± 1.73	DSB12	59.33 ± 0.58
14	DSG7	30.67 ± 1.53	DSB1	43.33 ± 1.53		
15	DSG8	31.00 ± 1.73	DSB2	45.67 ± 1.53		
16	DSB4	29.67 ± 1.53	DSB3	42.67 ± 0.58		
17	DSB7	30.00 ± 1.00	DSB6	42.67 ± 0.58		
18	DSB11	29.33 ± 1.53				
Mean ± SD		31.93 ± 2.60		44.59 ± 2.61		59.79 ± 2.49

*Mean ± SD.

The isolates also varied in their virulence demonstration during pathogenicity trials. Based on average lesion size, the isolates were divided into three virulence groups viz. Group I-least virulent which produced a canker lesion of < 100 mm length; Group II-moderately virulent which produced a lesion size of 100–150 mm length and Group III-highly virulent which produced a canker lesion of > 150 mm length (Table 4). Cankers show more extension longitudinally than radial expansion, for that reason canker length was used as a standard for comparing canker sizes. Group I accommodated 47.91 per cent of isolates (23 isolates) and was categorized as least virulent group. Group II accommodated 41.66 per cent isolates (20 isolates) and was considered as moderately virulent. Group III accommodated only 10.41 per cent of isolates (5 isolates) and was considered as highly virulent group (Table 4).

**Table 4:** Grouping based on virulence.

Group	Lesion size (mm)	Isolates	Percent isolates
Least virulent	< 100 mm	DSS2, DSS4, DSS5, DSS7, DSS11, DSS12, DSP1, DSP2, DSP4, DSP5, DSP6, DSP7, DSP8, DSG2, DSG3, DSG4, DSG6, DSG7, DSG8, DSB2, DSB4, DSB7, DSB11	47.91
Moderately virulent	100-150 mm	DSS1, DSS3, DSS6, DSS9, DSS10, DSP3, DSP9, DSP10, DSP11, DSP12, DSG5, DSG9, DSG11, DSG12, DSB1, DSB3, DSB6, DSB8, DSB9, DSB12	41.66
Highly virulent	> 150 mm	DSS8, DSB5, DSB10, DSG1, DSG10	10.41

### Genetic diversity

Initially 17 ISSR primers were screened for standardization using 10 randomly selected isolates of the pathogen. Among these 12 ISSR primers failed to produce polymorphic, reproducible, and scorable bands and hence did not yield any information. The remaining 5 primers resulted in polymorphic and reproducible bands, hence, were adopted for studying genetic diversity between the isolates of *D. seriata*. DNA fingerprinting of *D. seriata* isolates derived from diverse geographical locations clearly showed distinct banding pattern. The amplification products produced 65 bands in total ranging from 300 to 2000 bp, out of which 63 (96.92%) were polymorphic (Table 5).

**Table 5:** Annealing temperatures and output results for five ISSR primers used in the genetic diversity study of 48 isolates of *D. seriata.*

ISSR Primer	Annealing temperature (°C)	Amplified bands (*n*)	Polymorphic bands (*n*)	PBP (%)
HBH(AG)5	47	21	21	100.0
(CT)5	52	15	14	93.00
DDB(CCA)5	54	8	8	100.0
DBD(CA)5	47	10	10	100.0
DHB(CGA)5	54	11	10	90.90
Total	–	65	63	96.60

PBP (%) = polymorphic bands percentage.

Under similar experimental conditions, all the primers amplified the DNA from ten randomly chosen isolates while testing for reproducibility of the results. Out of 50 total combinations (10 isolates × 5 primers) band patterns identical to band arrangements in ISSR characterization were detected in 21 and the proportion of coincident bands exceeding 90% were observed in 35 combinations. Among all the combinations the coincident bands were estimated to be present with an overall percentage of 94%.

### Genetic variation analysis

**Figure 4:**
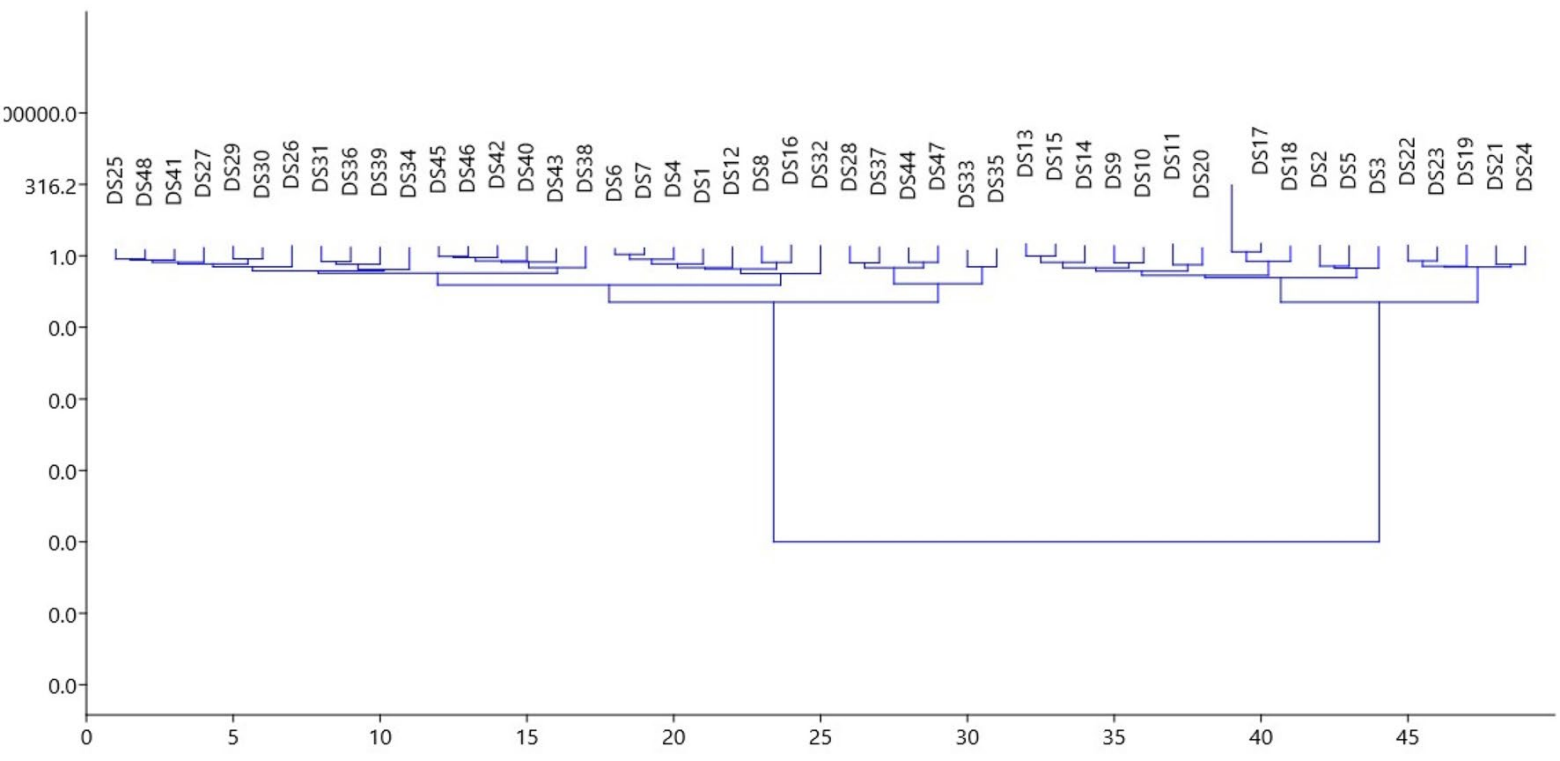
UPGMA based dendrogram showing genetic diversity among *D. seriata* isolates based on Jaccard’s coefficient of similarity using ISSR primers.

Four distinct clustering techniques were used to determine the most probable number of communities that the samples obtained would represent, assign individuals to these populations, and estimate the degrees of variance. Except for one isolate, i.e. DS38, which split as an independent lineage, the cluster analysis based on the unweighted Neighbor-joining approach yielded a dendrogram that grouped all the isolates representing four geographical populations into two primary clusters. The cluster first comprised of 26 isolates while as cluster second comprised of 21 isolates. The assignment of isolates into these two clusters was independent of their geographical background as both the clusters contained individuals from all the four geographical populations. Both the major clusters were further divided into smaller sub-clusters which were also independent of the geographical origin of the isolates. This demonstrates that genetic diversity exists both inside and among population isolates (Fig. 4).

The average number of observed alleles (Na) and effective alleles (Ne) across all the loci varied between 1.385 and 1.662 and 1.345 and 1.393, respectively, across the four distinct regional populations (PL, SH, BR, and GN). The highest number of observed alleles (1.662) were found in the population SH belonging to district Shopian of the valley, while as lowest number of observed alleles (1.345) were found in the population GN belonging to district Ganderbal, with mean value of 1.504. Again, the highest effective allele number (1.393) was found in the population SH while as the lowest number of effective alleles (1.345) were found in the population PL belonging to district Pulwama, with an average of 1.372. Similarly, the values of gene diversity (h) and unbiased gene diversity (uh) ranged from 0.218 to 0.242 and 0.238 to 0.264, respectively. The highest values of h (0.242) were recorded for the population SH pertaining to geographic locations of district Shopian and lowest values of h (0.218) were recorded for the population PL pertaining to geographic locations of district Pulwama, with an average of 0.228. Similarly, the highest values of uh (0.264) were recorded for the population SH belonging to geographic locations of district Shopian and lowest values (0.238) were recorded for the population PL, with an average of 0.248. Based on the Shannon’s information index (I), the highest diversity was recorded in the population SH (I = 0.377) and lowest diversity was recorded in the population PL and GN (I = 0.341), with an average of 0.350. The percentage of polymorphic loci among the populations ranged from 66.15% in GN to 81.54% in SH with an average of 72.31% (Table 6).

**Table 6:** Genetic diversity of four populations of *D. seriata* based on ISSR profiling.

Population	N	Ppl	Na	Ne	I	h	uh
PL	12	73.85%	1.554 ± 0.098*	1.345 ± 0.039	0.341 ± 0.030	0.218 ± 0.021	0.238 ± 0.023
SH	12	81.54%	1.662 ± 0.091	1.393 ± 0.042	0.377 ± 0.029	0.242 ± 0.021	0.264 ± 0.023
BR	12	67.69%	1.415 ± 0.109	1.372 ± 0.043	0.343 ± 0.033	0.225 ± 0.023	0.246 ± 0.025
GN	12	66.15%	1.385 ± 0.111	1.380 ± 0.045	0.341 ± 0.034	0.226 ± 0.024	0.246 ± 0.026
Mean	12	72.31% ± 3.50%	1.504 ± 0.052	1.372 ± 0.021	0.350 ± 0.016	0.228 ± 0.011	0.248 ± 0.012

*N* Sample size, *Ppl* percentage of polymorphic loci, *Na* observed number of alleles, *Ne* effective number of alleles, *I* Shannon’s information index, *h* diversity, *uh* Unbiased diversity.

*The values after ± denote the standard errors.

The average genetic distance and identity of Nei among *D. seriata* populations was 0.018–0.023 and 0.977–0.982, respectively (Table 7). The BR population of Baramullah, the SH population of Shopian, and the GN population of Ganderbal were found to have the highest genetic differences. The SH populations of Shopian and the PL populations of Pulwama were found to have the least genetic distance. Likewise, the SH population of Shopian and the PL population of Pulwama demonstrated the highest levels of genetic similarity. Ganderbal's GN and Baramulla's BR populations had the fewest genetic characteristics. The findings of Nei's genetic distance analysis showed that groups that were geographically close to one another had the lowest genetic distances, whereas populations that originated in various locations had the greatest genetic distances.

**Table 7:** Nei’s genetic identity and genetic distance of five populations based on ISSR.

Population	PL	SH	BR	GN
PL		0.982	0.981	0.980
SH	0.018		0.978	0.978
BR	0.019	0.023		0.977
GN	0.021	0.022	0.023	

Genetic distance (below diagonal) and Nei’s genetic identity (above diagonal).

**Figure 5:**
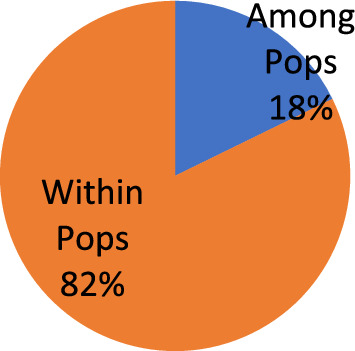
Analysis of molecular variance (AMOVA) of the binary data matrix performed in Genalex.

The geographically defined populations had considerable genetic diversity, according to the analysis of molecular variance (AMOVA). Although just 18% of the overall genetic diversity was found across populations from various geographical locations, most of the genetic variation (82%) was found within populations (among individual isolates) (Table 8; Fig. 5).

**Table 8:** Analysis of molecular variance (AMOVA) for testing homogeneity of genotype distribution in the *D. seriata* populations.

Source of variation	df	SS	MS	Estimated variance	Percentage (%)	PhiPT	*P* value
Among Pops	3	73.563	24.521	1.474	18%		
Within Pops	44	300.417	6.828	6.828	82%	0.178	0.010
Total	47	373.979		8.302	100%		

*df* degree of freedom, *SS* sum of squared observations, *MS* mean of squared observations, *PhiPT* proportion of the total genetic variance that is due to the variance among individuals within a variant.

**Figure 6:**
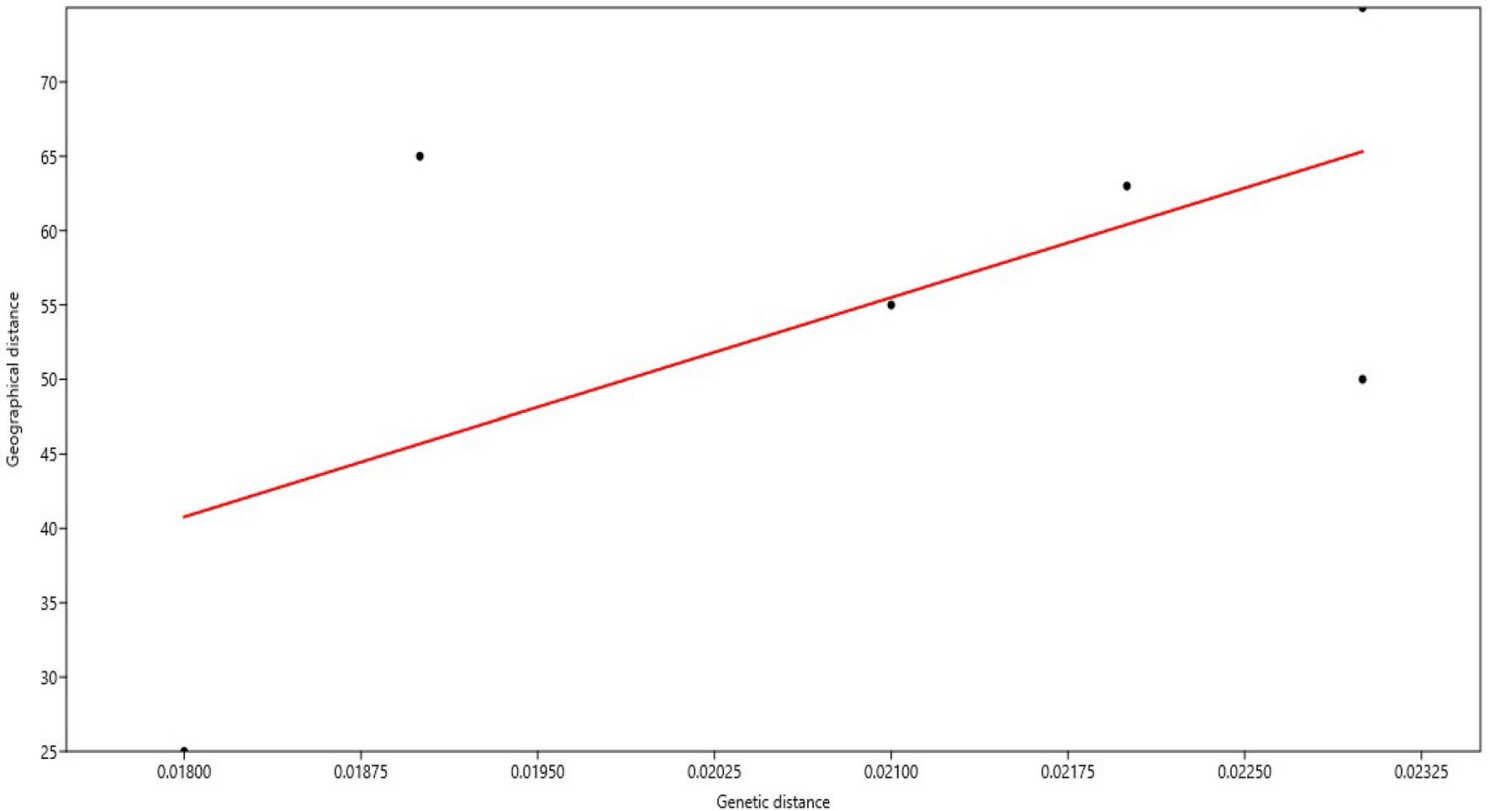
Correlation between genetic and geographic distances among 4 populations of *D. seriata*.

It was noted that there were statistically significant differences between the populations (*P* = 0.01). Pairwise PhiPT values estimated the degree of genetic divergence between the four populations. The average degree of genetic similarity was calculated to be 0.178 (average PhiPT value) (Table 9). The populations PL and GN showed the largest genetic difference (0.230), whereas the populations GN and BR showed the lowest genetic differentiation (0.076) (Table 9). The pairwise PhiPT values between the populations of South Kashmir (SH and PL; PhiPT = 0.103) were much lower than those between the populations of South and North Kashmir (PL-BR, SH-GN, and SH-BR), indicating that there was less genetic difference between these groups. The Mantel test confirmed any potential association between the geographic and genetic distances between the corresponding groups. The test's results revealed no connection between genetic and geographic distances (r = 0.591; p = 0.210) (Fig. 6).

**Table 9:** Pairwise population PhiPT values based on 999 permutations from AMOVA analysis among four populations.

Population	PL	SH	BR	GN
PL	–			
SH	0.103	–		
BR	0.173	0.228	–	
GN	0.230	0.223	0.076	–

**Figure 7:**
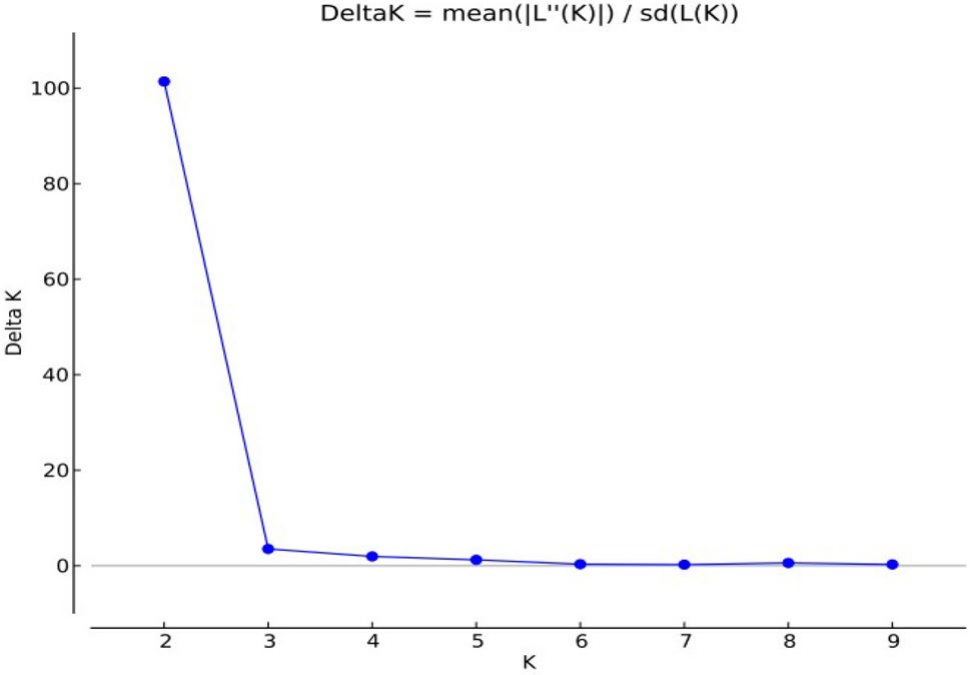
Result of Bayesian assignment analysis suggesting *K* = 2 as most likely number of clusters as delta *K* value was maximum at *K* = 2.

The analysis performed with the help of structure software divided the 48 *D. seriata* isolates into two genetically distinct clusters. The log likelihood values (Ln P(D)) increased from K = 1 to K = 2 and was maximum at K = 2 (ln *P*(*D*) = 2145·68). The standard deviation showed a slow increase from K = 1 to K = 2 and thereafter it showed a rapid increase from K = 3 to K = 10. This showed that the population structure of the *D. seriata* pathogen population is best explained by K = 2 clusters. This is further strengthened by the value of ∆*K* which was highest at K = 2 (∆*K* = 101·39) and after that it decreased at a very fast rate. The highest value of ∆*K* that determines the best fit of *K* was derived by employing STRUCTURE HARVESTER (Fig. 7).

**Figure 8:**
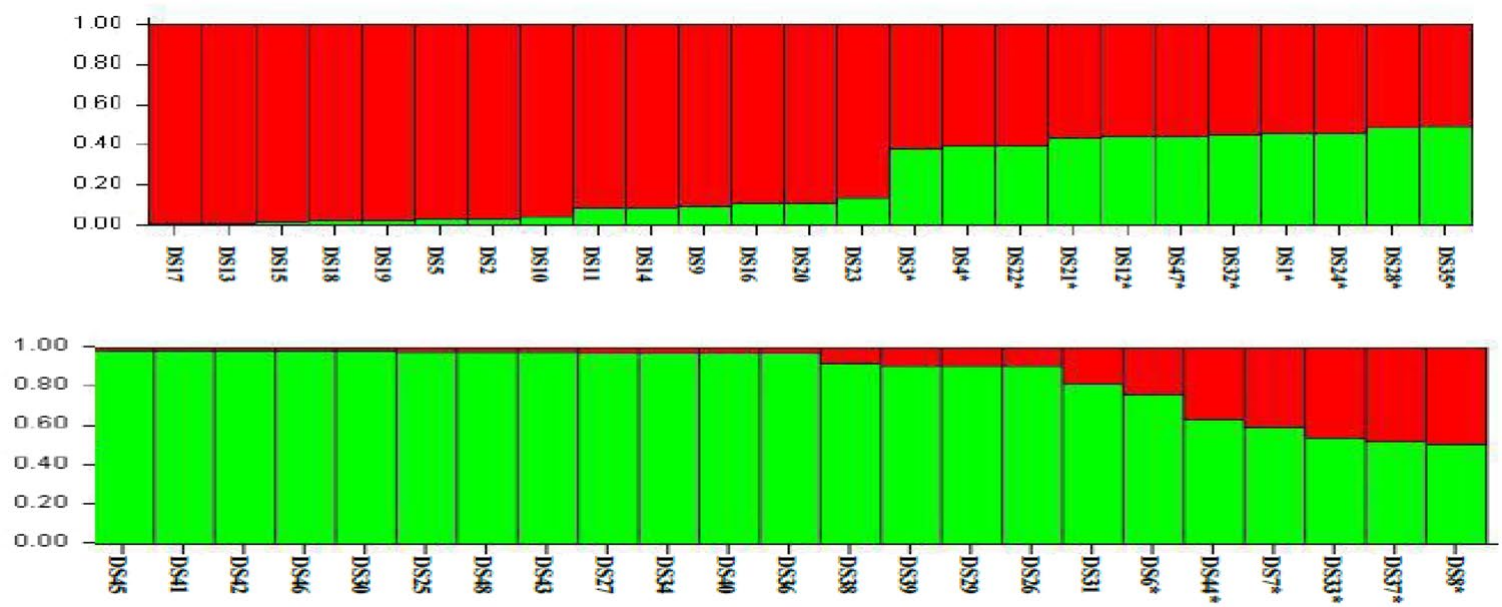
Estimated genetic structure for K = 2 groups of 48 *Diplodia seriata* isolates inferred by Bayesian clustering of ISSR data in 20 independent runs using the software STRUCTURE v. 2.2.3. Each isolate is represented by a bicolored column according to its posterior probability.

The clusters formed in the structure analysis are shown in the figure below. Most of the individual isolates were find out to possess 80% of their heredity from one of the two distinct populations (Fig. 8), while as 17 isolates were found to have mixed ancestry with less than 80% posterior probability to any of the two clusters. The cluster in red consisted of 14 *D. seriata* isolates while as the cluster in green consisted of 17 isolates. The distribution of isolates into different clusters was irrespective of their geographical origin, hence both the clusters contained isolates from all the four predetermined geographical populations.

**Figure 9:**
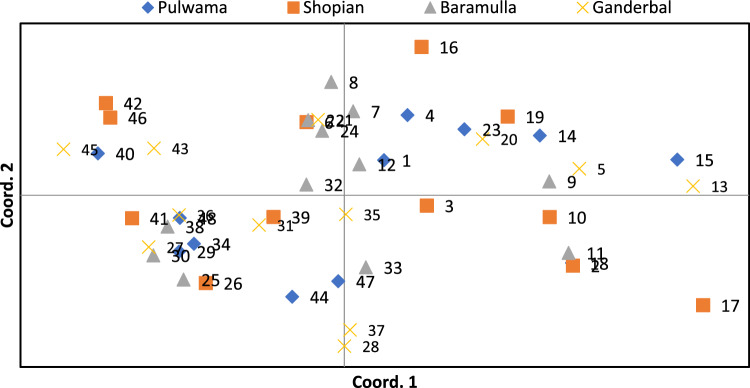
Principal coordinate analysis of four populations of *D. seriata* based on ISSR molecular marker analysis.

The principal coordinate analysis (PCoA) was carried out with Genalex software on the whole data set to verify the consistency of genetic differentiation clustering performed by different clustering methods. The two-dimensional PCoA plot revealed that 15.45% and 9.20% of the total genetic variation is represented by the coordinate 1 and coordinate 2, respectively. It is evident from the PCoA plot that except two individuals as outliers and two small groups were formed, and all the other individuals formed a common group, however the individuals in all the groups were intermixed (Fig. 9).

**Figure 10:**
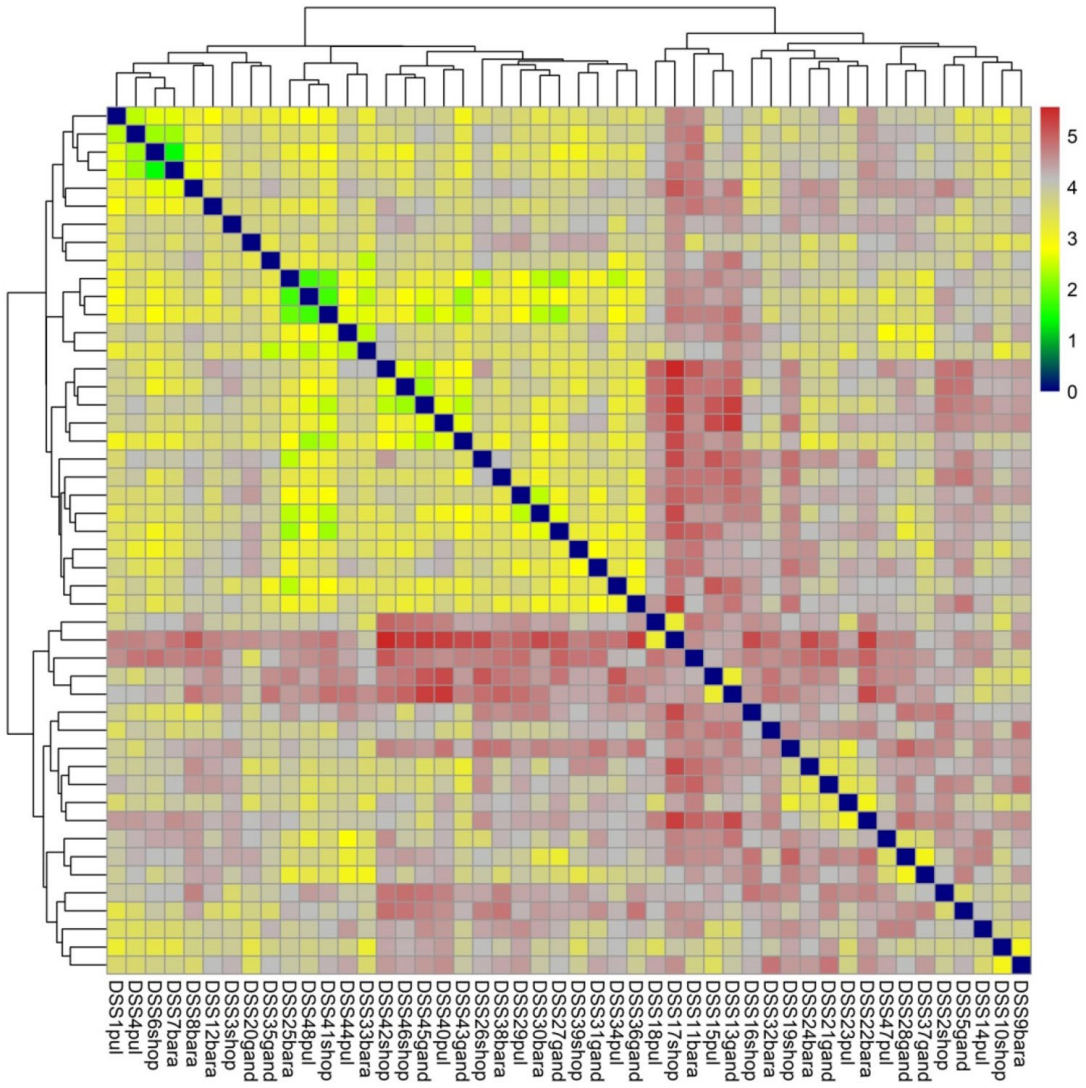
Heat Map of isolates. The 48 fungal isolates representing different geographical regions were used to construct a heatmap. Heatmap shows that samples cluster irrespective of the geographical origin. Clustering demonstrated that all the isolates grouped into two main clusters which is consistent with the clustering by STRUCTURE.

This shows that the PCoA clustering was independent of the geographical origin of the isolates which is in accordance with other clustering analysis methodologies. The heatmap constructed with R software also divided the population of all the isolates into two major clusters which are further subdivided into sub-clusters. It is clear from the heatmap that the genetic variation is spread among the major clusters and sub-clusters irrespective of the geographical origin of the individual isolates (Fig. 10).

## Discussion

In the present investigation, the phenotypic traits of the fungal isolates collected from apple black rot canker were in complete accord with those of the genus *Dilpodia*^43^. Depending on the color and septation of their conidia, the species of *Diplodia* may be roughly classified into two distinct groups based on morphological descriptions^44^. Most *Diplodia* species, including *D. malorum*, *D. mutila*, *D. corticola*, *D. africana*, and *D. rosulata*, have aseptate, thick-walled conidia that may stay hyaline for a long time before becoming brown^43,45–48^. Nevertheless, in other species, including *D. alatafructa*, *D. pinea*, *D. scrobiculata*, *D. seriata*, and *D. pseudoseriata*, the conidia become colored before discharge from the pycnidia and mostly stay non-septate^43,44^. All of the isolates morphological characteristics in this investigation were consistent with those previously described for *D. seriata*^1,13^. Additionally, no significant differences were identified across regional or genetic groups.

Each of the isolates produced characteristic black rot canker symptoms on potted apple trees and the isolates were recovered from 98% of the inoculated apple twigs, hence proving the isolates to be potential pathogens of apple. *Botryosphaeriaceae* fungi being cosmopolitan in nature^13^ have been reported to be common pathogens of a vast diversity of woody hosts including apple^49–51^. Pathogenicity of *D. seriata* has been evaluated on shoots, fruits, and leaves of apple in some countries^23,24^. The experimental data on the pathogenic nature of *Diplodia* spp. on woody plants is contentious. For instance, *D. seriata* is regarded as a secondary and weak pathogen of apples in England and New Zealand^27^, while it is reported as highly pathogenic on USA apples where it is responsible for apple cankers, leaf spots and fruit rot^14,28,29^. Similarly, *D. seriata*, being highly virulent on grapevines has been considered as a primary and virulent pathogen^31,33^ but other workers have observed it to be saprophyte or weak pathogen on grapevines^6,36,43^. These contradictions in pathogenicity may be due to differences in virulence among the isolates. However, in the present investigation all the isolates of *D. seriata* were found to be highly pathogenic to apple as reflected by the length of lesions produced on the inoculated apple seedlings which were much larger than the lesions in control.

A significant variation was observed in isolates of *D. seriata* with respect to lesion length on artificially inoculated apple trees of cultivar Red Delicious. The variation in the length of canker lesions caused by different isolates under pathogenicity tests confirms the existence of virulence variability among the isolates of *D. seriata*. The isolates were categorized into 3 virulence groups depending on average canker length. The isolates showed no relevance between the virulence and geographical origin of isolates which was later supported by genetic grouping as well. Based on length of lesions produced in pathogenicity tests on grapevine canes, the *D. seriata* isolates were divided into four virulence groups^31^. Variation in *D. seriata* isolates with reference to wood colonization on grapevines has also been reported^6^. Similar findings on the variability in virulence of *D. seriata* have been recorded by other workers as well^30,32^. While discussing the contrasting reports regarding the pathogenicity of *D. seriata*, Savocchia et al.^33^ concluded that these differences may be because of the presence of intraspecific variations within this fungus. Our findings are well supported by the observations of Elena et al.^12^ who reported “no relationship between the genetic groups and the virulence of isolates of *D. seriata* on grapevine”. Similar conclusions were drawn by other workers e.g., Billones-Baaijens et al.^41^ and Baskarathevan et al.^40^ who observed no relationship between genotypic and pathogenic variability in the isolates *Neofusicoccum luteum* and *N. parvum,* respectively. Again, no correlation was found to exist between the radial mycelial growth and the virulence variability of isolates because the isolates which showed maximum radial mycelial growth did not always produce longest cankers as it should have been. This could be attributed to the reasons like production of phytotoxic secondary metabolites by *D. seriata* as reported by Martos et al.^52^, or presence of some other mechanism besides fungus mycelium growth inside the host which may be influencing the virulence of the pathogen^12^.

The ISSR technique proved effective for ascertaining the genetic diversity between the isolates of *D. seriata* having diverse geographical origins. Five ISSR primers recorded a high degree of polymorphism in the isolates of *D. seriata*, hence proving that ISSR markers are very relevant in verifying the genetic diversity in this fungus. The ISSR markers have been found relevant for investigating the genetic variability in *D. seriata* and other fungal plant pathogens by various other workers^12,54,56–59^.

For investigating the population structure of *D. seriata* isolates Bayesian clustering of ISSR markers proved very useful and provided the basis to investigate the genetic structure. The analyses performed with STRUCTURE divided the isolates into two main clusters however; it was observed that some of the isolates did not constantly belong to these clusters. Further, the isolates originating from diverse geographical regions did not fall in different genetic groups, indicating that the isolates belong to a common ancestral background irrespective of their geographical origin. Elena et al.^12^, reported high genetic diversity in *D. seriata* on grapevine from Spain using ISSR markers and recorded that there was no relation between the clustering groups and the geographic origin of isolates. While performing ITS sequencing of *D. seriata* isolates Phillips et al*.*^13^ concluded that no correlation existed between the clustering structure and the geographical origin of the isolates.

Clustering of *D. seriata* isolates at regional level revealed that most of the variability was found within groups than between the groups. This indicates that the genetic diversity between the geographic populations is less but within populations diversity is high. Padder et al.^56^ reported high genetic diversity in *Venturia inaequalis* populations on apple in Kashmir valley using ISSR markers and found greater variability within populations than among populations. Dinolfo et al.^57^ studied intraspecific diversity within the isolates *Fusarium poae* derived from Argentina and England but there was no definite relation between genetic diversity and geographic origin, and diversity was high within rather than between the Argentinean and English populations. Fan et al.^55^ analyzed genetic variation in Chinese and Californian isolates of *Monilinia fructicola* using ISSR markers and found that the diversity was high within population rather than between populations. Many other researchers have also reported high genetic diversity in fungal plant pathogens using ISSR markers^58^. Although only two clusters were derived in the STRUCTURE analysis of the data generated from the 48 isolates of *D. seriata*, the ISSR technique revealed a high level of polymorphism (96%). Similar results were recorded by Elena et al.^12^ who reported 88% polymorphism in 83 *D. seriata* isolates from Spain. Employing AFLP markers Martin^38^ recorded 86% polymorphism in the isolates of *D. seriata* derived from Spain. With UP-PCR technique Billones-Baaijens et al.^41^ recorded 93% polymorphism in the isolates of *N. luteum*. Using the same technique, Baskarathevan et al.^40^ also recorded 93% polymorphism in 60 isolates of *N. parvum*. Many factors such as gene flow, recombination (sexual or parasexual), mutation and adaptation of the pathogen to diverse host species may be responsible for the presence of high genetic diversity in *D. seriata*. Although Hesler recorded the formation of perfect state (*Botryosphaira obtusa*) for the smoky canker pathogen on apple twigs but other researchers from India and other countries failed to report any sexual state in field^22^. Thus, further investigations are required to be undertaken both under natural field conditions and at genetic levels to verify whether some hidden telomorphic state is responsible for the high genetic diversity in this fungal species. Estimates of pairwise *F*_*ST*_ were low in geographical populations. This indicates that there is frequent gene flow between populations probably due to movement of infected planting material.

## Conclusions

The morphological differences shown by the isolates in phenotypic characterization did not show any correlation with the genetic clusters. Moreover, the pathogenicity tests revealed *D. seriata* as highly pathogenic on apple and indicated variability in virulence, but the pathogenic variability was not correlated to either the genetic variability or the geographical origin of the isolates. To the best of our knowledge, this combined phenotypic, pathogenic, and genetic variability of *D. seriata* isolates causing black rot canker, dieback, and fruit rot of apple in India is studied for the first time and it will help in understanding the pathogenic nature of the *D. seriata* populations and development of more effective management strategies in future.

## Materials and methods

### Fungal isolates

The disease samples collected from different parts of the apple trees during the years 2016 and 2017 were cleaned of by using distilled water. The bark at the diseased portions was carefully removed and the area was surface sterilized with 70% ethanol. Wood sections (5 mm^2^) were taken along the margins of canker lesions by using sterilized blade and the tissue bits were sterilized for 60 s in 1% sodium hypochlorite solution. The bits were washed thrice with sterilized distilled water to eliminate the remains of chemical followed by drying on blotting paper and inoculated on acidified PDA. The inoculated plates were placed under incubation at 25 ± 1 °C in the darkness. All the isolates of *D. seriata* collected from different geographical areas were purified by single spore technique^60^. *D. seriata* was identified based on morphological characters as per the descriptions of fungus given by Phillips^13^. In all, 48 isolates (Table 10) of *D. seriata* having different geographical origins were maintained for further studies and the stock cultures were preserved under refrigeration at 4 °C inside the refrigerator.

**Table 10:** Isolate No., geographical location, genetic grouping (structure) and other details of *D. seriata* isolates obtained from the host *Malus* × *domestica*.

Isolate	Locality	District	structure group (K = 2)	Other studies	Geographical coordinates	Elevation (m)
DS1	Bellow	Pulwama	1*	1	33.8348° N, 74.8588° E	1638
DS2	Imam sahib	Shopian	1	NS	33.7441° N, 74.9106° E	1995
DS3	Imam sahib	Shopian	1*	NS	33.7441° N, 74.9106° E	1995
DS4	Rajpora	Pulwama	1*	1	33.8243° N, 74.8531° E	1800
DS5	Lar	Ganderbal	1	NS	34.2620° N, 74.7550° E	1650
DS6	Pinjoora	Shopian	2*	NS	33.7394° N, 74.8646° E	1594
DS7	Sopore	Baramulla	2*	NS	34.2986° N, 74.4701° E	1582
DS8	Rafiabad	Baramulla	2*	1	34.2111° N, 74.3408° E	1577
DS9	Rafiabad	Baramulla	1	NS	﻿34.2111° N, 74.3408° E	1577
DS10	Kaprin	Shopian	1	NS	33.6645° N, 74.8962° E	1739
DS11	Pattan	Baramulla	1	NS	34.1541° N, 74.5603° E	1553
DS12	Pattan	Baramulla	1*	1	34.1541° N, 74.5603° E	1553
DS13	Gotli bagh	Ganderbal	1	NS	34.2800° N, 74.8347° E	1592
DS14	Bellow	Pulwama	1	NS	33.8348° N, 74.8588° E	1638
DS15	Rajpora	Pulwama	1	NS	33.8243° N, 74.8531° E	1800
DS16	Kaprin	Shopian	1	1	33.6645° N, 74.8962° E	1739
DS17	Imam sahib	Shopian	1	NS	33.7441° N, 74.9106° E	1995
DS18	Keller	Pulwama	1	NS	33.8997° N, 74.7455° E	1739
DS19	Pinjoora	Shopian	1	1	33.7394° N, 74.8646° E	1594
DS20	Lar	Ganderbal	1	NS	34.2620° N, 74.7550° E	1650
DS21	Zazuna	Ganderbal	1*	NS	34.2306° N, 74.6854° E	1619
DS22	Pattan	Baramulla	1*	NS	34.1541° N, 74.5603° E	1553
DS23	Rajpora	Pulwama	1	NS	33.8243° N, 74.8531° E	1800
DS24	Sopore	Baramulla	1*	NS	34.2986° N,, 74.4701° E	1582
DS25	Sopore	Baramulla	2	NS	34.2986° N,, 74.4701° E	1582
DS26	Kaprin	Shopian	2	1	33.6645° N, 74.8962° E	1739
DS27	Zazuna	Ganderbal	2	NS	34.2306° N, 74.6854° E	1619
DS28	Zazuna	Ganderbal	1*	NS	34.2306° N, 74.6854° E	1619
DS29	Bellow	Pulwama	2	NS	33.8348° N, 74.8588° E	1638
DS30	Rafiabad	Baramulla	2	NS	﻿34.2111° N, 74.3408° E	1577
DS31	Gotli bagh	Ganderbal	2	1	34.2800° N, 74.8347° E	1592
DS32	Pattan	Baramulla	1*	NS	34.1541° N, 74.5603° E	1553
DS33	Rafiabad	Baramulla	2*	NS	34.2111° N, 74.3408° E	1577
DS34	Keller	Pulwama	2	NS	33.8997° N, 74.7455° E	1739
DS35	Lar	Ganderbal	1*	NS	34.2620° N, 74.7550° E	1650
DS36	Zazuna	Ganderbal	2	NS	34.2306° N, 74.6854° E	1619
DS37	Gotli bagh	Ganderbal	2*	NS	34.2800° N, 74.8347° E	1592
DS38	Sopore	Baramulla	2	NS	34.2986° N, 74.4701° E	1582
DS39	Imam sahib	Shopian	2	NS	33.7441° N, 74.9106° E	1995
DS40	Rajpora	Pulwama	2	NS	33.8243° N, 74.8531° E	1800
DS41	Pinjoora	Shopian	2	1	33.7394° N, 74.8646° E	1594
DS42	Pinjoora	Shopian	2	NS	33.7394° N, 74.8646° E	1594
DS43	Gotli bagh	Ganderbal	2	NS	34.2800° N, 74.8347° E	1592
DS44	Bellow	Pulwama	2*	NS	33.8348° N, 74.8588° E	1638
DS45	Lar	Ganderbal	2	1	34.2620° N, 74.7550° E	1650
DS46	Kaprin	Shopian	2	NS	33.6645° N, 74.8962° E	1739
DS47	Keller	Pulwama	1*	NS	33.8997° N 74.7455° E	1739
DS48	Keller	Pulwama	2	NS	33.8997° N 74.7455° E	1739

Numbers with an asterisk are indicative of isolates with < 80% assignment probability to different groups. Other studies: 1 represents the isolates selected for verifying the reproducibility of ISSR technique.

*NS* not selected.

### Pathogenicity tests

Pathogenic variability was studied by performing pathogenicity tests of all the 48 isolates on healthy two-year-old potted apple seedlings (cv. Red Delicious grafted on Quince root stock) following the procedure adopted by Spiers^61^. The selected plants were first treated with copper oxychloride 50 WP (Blitox 50) @ 0.3% as a spray to eliminate the existence of any pathogens which may be present on the host surface. The pots were kept inside a polyhouse and timely irrigated to prevent the young apple seedlings from water stress and intermittently sprayed with distilled sterilized water to maintain high humidity inside the chambers. The inoculations were done on dormant apple twigs. Each isolate was inoculated on three twigs and the tests were performed as per the following procedure.

On the selected twigs, after surface sterilizing the designated area with 70% ethanol, a 5 mm horizontal cut was given cutting the bark of the twig. Starting from the mid-point of the first cut, another vertical cut of 5 mm was made. The bark of this T-shaped cut was loosened with the help of a sterilized scalpel. A mycelial disc (4 mm) was put beneath the bark of incision and a cotton swab moistened with sterilized water was placed on the inoculated cut followed by wrapping with adhesive paper tape. The control was inoculated with plain media (PDA) disc and the incisions were covered as described earlier. The pathogenicity tests were regularly tracked for canker formation. Observations in terms of symptom development and lesion size by different isolates were recorded. The pathogen was re-isolated from the inoculated twigs to fulfill Koch's postulates. For re-isolations 1 cm pieces of wood after removing bark were cut at the two ends of canker lesions and sterilized in ethanol (70%) for 4 min, blotted dry and inoculated on Petri plates containing acidified PDA. The plates were kept under incubation at 25 ± 1 °C and observed for morphological characters of *D. seriata*.

### Phenotypic characterization

#### Radial mycelial growth

All the 48 isolates of *D. seriata* from the four geographical locations were included in this study. Each Petri plate containing PDA was inoculated in the center with 5 mm culture bit of the actively growing isolate of *D. seriata* followed by incubation of the plates at 25 ± 1 °C. Each isolate was grown on four PDA plates. The observations on radial mycelial growth were recorded after 72 h by taking average of four radial measurements perpendicular to each other. The test was conducted thrice for the purpose of accuracy.

#### Conidial size and shape

Conidial characteristics such as shape, size, color and septation of all the 48 isolates were assessed on oatmeal agar medium. The cultures were kept on a bench in the laboratory for 4 weeks at about 20–25 °C for inducing pycnidia formation under diffused sunlight. The spore measurements of each isolate were recorded under light microscope [HF (bright field, filter 1); Carl Zeiss] at 100× magnification by preparing semi-permanent slides in lactophenol under 10 random microscopic fields after calibrating the microscope following standard procedures^64^. For each isolate the conidial dimensions (length and width) of 50 conidia were recorded, and the length: width ratio was estimated.

### Genomic DNA extraction and PCR amplifications

For raising enough mycelium for DNA extraction, all the isolates of *D. seriata* were grown on PDA broth in Erlenmeyer flasks. Mycelium was harvested by filtering the contents of the flask through a double layered filter paper, dried and stored at − 80 °C. Adopting modified CTAB (Cetyl trimethyl ammonium bromide) method^62^, total genomic DNA was extracted from all the isolates. The DNA was kept at 4° C for amplification and stored at − 20° C for future use.

A set of 17 ISSR primers namely (CAG)5, (ACA)5, BDB(ACA)5, (AG)5, (AT)8B, (CA)5, (GA)5YC, (TC)8RA, (CCA)5, (CGA)5, HBH(AG)7, (CT)5, (TG)5, (AG)5YC, (AG)5YA, (TG)8RC and BBD(AAC)5 were chosen for conducting preliminary screening against 10 randomly selected isolates to study the polymorphism. Five primers which were consistent in polymorphism during preliminary screening were selected for ISSR characterization of all the 48 isolates of *D. seriata*. PCR amplifications were performed in 0.2 ml PCR tubes in a T-Gradient Whatman Biometra thermal cycler with 50–60 ng of genomic DNA per isolate. The final volume of PCR mix was adjusted to 25 μl per reaction with RNAse-free water.

The reaction mixture in PCR tubes were given short spin or vortexed in microfuge and placed in 96-well thermal cycler. PCR amplifications were conducted in thermal cycler programmed for initial step at 94 °C for five minutes followed by 35 denaturation cycles at 94 °C for one minute, annealing at appropriate temperature (Table 11) for one minute, elongation at 72 °C for two minutes and a final step of extension for 10 min. To 25 μl of the amplified PCR product, 5 μl of 6X loading dye was added to have final concentration of 1X.

**Table 11:** ISSR Primers, their sequences and annealing temperatures for PCR analysis of different isolates of *D. seriata.*

S. no.	Primer	Sequence	Annealing temperature
1	(AG)5	5′ AGAGAGAGAG 3′	47 °C
2	(CA)5	5′ DBDCACACACACA 3′	47 °C
3	(CCA)5	5′ DDBCCACCACCACCACCA 3′	54 °C
4	(CGA)5	5′ DHBCGACGACGACGACGA 3′	54 °C
5	(CT)5	5′ CTCTCTCTCTCTCTC 3′	52 °C

The quality of PCR products was evaluated on 2% agarose gel. TAE (0.5X) was used to prepare the gel and ethidium bromide at 0.5 μg/μl was added to it. The gel was loaded with amplified products of all the 48 isolates of *D. seriata* and run at 5 V/cm and visualized under UV light. The photograph of resultant banding pattern was taken using Alfa Imager gel documentation system. The size of ISSR alleles was determined by their relative positions with respect to DNA ladder (100 base pair). The amplification reactions of all the primers were repeated thrice for a single DNA extraction of each of the isolates. Further, for band scoring only clear and reproducible bands were taken into consideration.

### ISSR characterization and analysis of genetic variability

The banding pattern of each primer was scored as 1 (band present) and 0 (band absent) and the pattern was converted into a binary matrix with the help of MS Excel. The binary data matrix was used to work out TNB (total number of bands), NPB (number of polymorphic bands), and PPB (percentage of polymorphic bands). The Jaccard’s coefficient of similarity was calculated in the PAST (v 4.0) software by making pairwise comparisons between the individual isolates. Furthermore, a dendrogram was constructed with the help of same software by adopting the UPGMA method. The cophenetic correlation coefficient was worked out by the PAST program for measuring the goodness of fit between the cophenetic matrix and the distance matrix^69^. For each marker the alleles were calculated in all the isolates by numbering the alleles as 1, 2, 3 and so on, starting from the allele possessing maximum molecular weight. The bands were visually scored as 0 (absent) and 1 (present). The binary data generated by ISSR primers was analyzed by cluster analysis utilizing the program NTSYS-pc version 2.02^63^. Based on shared bands among the isolates, similarity was estimated from the data generated by all the 5 ISSR primers using Jaccard’s coefficient. The NTSYS software was utilized for determining the Nei and Li^65^ coefficients to ascertain the similarity between the isolates of *D. seriata*. The investigation of genetic diversity among the isolates of *D. seriata* was done by analysis of the band arrangements using a Bayesian clustering approach executed in STRUCTURE v.2.2.3^67^ considering a population admixture model wherein within population correlations of allele frequencies are worked out. The runs for each K value (1 to 10) were set for 20 independent replications having a burn‐in period of 100,000 accompanied by 500,000 iterations of the Markov Chain Monte Carlo (MCMC). The posterior probability of the data (ln P(D)) was estimated utilizing the ad hoc ΔK statistic, based on rate of change between successive K values, and this estimate was later employed for inferring the uppermost level of structure in the dataset^67^. The highest values of Ln P(D), which represents the posterior probability of the data for a certain K, and ΔK were used to determine K (the rate of change in the log probability of data between successive values of K)^76^. The sample populations were divided into genetic groupings using bar graphs that represent individual assignment probability. By using Structure Harvester v6.0 to generate the K values, the best-fit population cluster number (K) was verified^76^. Employing ARLEQUIN v. 3.5^68^, AMOVA was used to find out the differences in assigning the isolates to different clusters which were obtained after Bayesian analyses.

Further, the pairwise population differentiation (PhiPT) was also worked out by utilizing Genalex 6.503 package^69–71^. Microsatellite data set were analyzed for basic genetic diversity indices such as, N: Sample size; Ppl: percentage of polymorphic loci; Na: observed number of alleles; Ne: effective number of alleles; I: Shannon’s information index; h: diversity; uh: Unbiased diversity using population genetic software GenALex 6.503^71^.

The principal coordinate analysis (PCoA) was conducted by employing Genalex software to generate a coordinate graph of the most significant axes to verify the clustering pattern. Distribution of the genetic variation into two components viz. within populations and among the populations was calculated by performing the analysis of molecular variance (AMOVA) in Genalex at 999 permutations.

### Statistical analysis

To estimate the population genetic structure and examine the distribution of genetic diversity, the isolates of *D. seriata* were primarily grouped into populations based on geographical origin. The pathogenicity experiments were conducted following randomized complete block design under poly house conditions. Groups based on geographic origin and those generated by the STRUCTURE analysis were considered as independent variables. ANOVA (One‐way) and posterior Tukey's Honestly Significant Difference tests were performed on dependent variables to find out the differences between the mean values. The molecular data generated during the study was analyzed with NTSYS- 2.02e; Arlequin v. 3.5; GenAlex 6.503; PAST 4.0 and STRUCTURE v. 2.2.3 as described earlier. The remaining data was analyzed by SPSS (ver. 18) and SAS (ver. 9.2) software.

## Data Availability

All data are within the manuscript.
